# Metformin and Gegen Qinlian Decoction boost islet α-cell proliferation of the STZ induced diabetic rats

**DOI:** 10.1186/s12906-022-03674-2

**Published:** 2022-07-20

**Authors:** Li Xu, Shreyas Jois, Hongliang Cui

**Affiliations:** 1 Medical experiment center, Shaanxi University of Chinese Medicine, Xianyang, 712046 China; 2 Toxicology Centre, University of Saskatchewan, Saskatoon, S7N 5B3 Canada

**Keywords:** Diabetes, Gegen Qinlian Decoction, Islet α-cells, Islet β-cells, Metformin

## Abstract

**Background:**

The traditional Chinese medicine Gegen Qinlian Decoction (GQD), as well as metformin, had been reported with anti-diabetic effects in clinical practice.

**Objective:**

To verify whether these two medicines effectively ameliorate hyperglycemia caused by deficiency of islet β-cell mass which occurs in both type 1 and type 2 diabetes.

**Methods:**

SD rats were injected with a single dose of STZ (55 mg/kg) to induce β-cell destruction. The rats were then divided into control, diabetes, GQD and metformin group. GQD and metformin groups were administered with GQD extract or metformin for 6 weeks. The islet α-cell or β-cell mass changes were tested by immunohistochemical and immunofluorescent staining. The potential targets and mechanisms of GQD and metformin on cell proliferation were tested using in silico network pharmacology. Real-time PCR was performed to test the expression of islet cells related genes and targets related genes.

**Results:**

Both GQD and metformin did not significantly reduce the FBG level caused by β-cell mass reduction, but alleviated liver and pancreas histopathology. Both GQD and metformin did not change the insulin positive cell mass but increased α-cell proliferation of the diabetic rats. Gene expression analysis showed that GQD and metformin significantly increased the targets gene cyclin-dependent kinase 4 (Cdk4) and insulin receptor substrate (Irs1) level.

**Conclusion:**

This research indicates that GQD and metformin significantly increased the α-cell proliferation of β-cell deficiency induced diabetic rats by restorin*g* Cdk4 and Irs1 gene expression.

## Background

There are about 422 million people have diabetes worldwide and approximately 1.5 million deaths were attributed to diabetes each year [1]. Due to diet and lifestyle changes, the incidence of diabetes is increasing exponentially. It was estimated that there will be nearly 550 million diabetic patients worldwide by 2030 [2]. Even though researchers have worked on diabetes for decades and improvised medicines are available on the market, its pathogenesis and development mechanism are not fully illustrated. A recent study suggested that islet β-cell mass is closely related to both type 1 diabetes (T1D) and type 2 diabetes (T2D) pathogenesis [3]. Islet β-cell mass decline reflects the onset of the T1D [4]. In T2D patients, islet β-cell mass was significantly decreased compared to the non-diabetic patients [5, 6] and β-cell mass deficit increased as the disease continues [7]. After the onset of diabetes, both T1D and T2D may cause massive destruction of islet β-cells symptoms [8]. Moreover, defect in β-cell mass is critical for the development of hyperglycemia in T2D [9]. Current research indicates that the death or dysfunction of islet β-cells is an important common marker for the development and progression of both types of diabetes [10, 11]. Streptozotocin (STZ) is an antibiotic that can produce islet β-cell destruction and is commonly used for establishing diabetic animal models [12]. By injecting STZ to animals with different doses or frequencies, it can induce T1D or T2D animal model [13].

Gegen Qilian Decoction (GQD) is a classic traditional Chinese medicine (TCM) formula of Zhongjing Zhang’s “Shang Han Lun” from 202 BC-220 AD [14]. It consists of 4 kinds of herbal medicines: *Pueraria lobate* (Willd.) Ohwi, *Coptis Chinensis* Franch, *Scutellaria baicalensis* Georgi and *Glycyrrhiza uralensis* Fisch [15]. Traditionally, GQD is used for diarrhea, in recent years it has been reported in many literatures for its ability to reduce blood glucose, insulin resistance and protective effects on diabetes related complications in both T2D patients and animals [15–19]. Due to its efficient and safe trait in glycemic control, GQD has been used in T2D therapy for many years [17]. However, there is insufficient evidence for the influence of GQD on islet cells, specifically, it is unclear if GQD is still effective to reduce blood glucose when lacking islet β-cells.

Metformin is a common prescription for T2D patients and is recommended by most diabetes associations [20]. Although metformin has been used for more than 30 years, the precise mechanism of how metformin reduces blood glucose remains controversial. Metformin enhances the action of insulin in the liver and skeletal muscle, therefore reducing insulin requirement for T2D patients [21]. It can effectively reduce hepatic gluconeogenesis resulting in a rapid lowering of blood glucose [22]. A previous study had proved that metformin suppresses gluconeogenesis by inhibiting mitochondrial glycerophosphate dehydrogenase [23]. Recently, it was reported that metformin enhances glucose uptake by regulating glucose transporter-4 endocytosis [24]. Although it is already known that metformin does not promote insulin secretion, whether it can act on other cells of the pancreatic islet is still unclear.

Even though, increasing evidence support to characterize both T1D and T2D by deficits in β-cell mass [25]. The effects of glucose-lowering medicine GQD and metformin on β-cell mass deficiency caused hyperglycemia is still elusive. In this study, the impact of GQD or metformin on STZ induced diabetic rats was investigated at physiological, immunohistochemical and molecular levels.

## Methods

### Animals and experimental groups

Male SD rats (200-240 g) were purchased from Experimental Animal Center of Xi’an Jiaotong University (SCXK 2012–003). Animals were kept at 23 ± 2 °C, 55 ± 5% relative humidity, 12 h/12 h light/dark cycle and free access to standard chow and drinking water. All animal experiments were carried out in accordance with the National Institutes of Health guide for the care and use of laboratory animals [26]. All experiments were performed under the guidelines of Experimental Animal Ethics Committee of Shaanxi University of Chinese Medicine (SUCMDL20190408010).

After 1 week of acclimatization, 40 rats were fasted for 12 h and then subjected to treatments. For the test group, 30 rats were randomly selected and injected intra-peritoneally with STZ (Solarbio-S8050, Beijing) at a dose of 55 mg/kg. For the control group, 10 rats were injected with an equal dose of 0.1 M sodium citrate buffer (pH = 4.4). After 72 h, if the fasting blood glucose (FBG) of the test group was ≥16.7 mmol/L, then the diabetic rat model was successfully established. This method was modified from Goyal et al. [27]. Finally, 24 active rats with FBG ≥ 16.7 mmol/L were randomly divided into the diabetes group, GQD group and metformin group, with 8 animals in each group. The animal food was weighted every day to calculate the average food intake.

### Gegen Qilian Decoction preparation and medicine administration

Herbs for GQD extract *Pueraria lobate* (Willd.) Ohwi (Lot No.: 140201, Guangxi), *Coptis Chinensis* Franch (Lot No.: 20101001, Sichuan), *Scutellaria baicalensis* Georgi (Lot No.: 131191, Hebei) and *Glycyrrhiza uralensis* Fisch (Lot No.: 20111001, Neimenggu) were provided by Shaanxi University of Chinese Medicine school hospital. The voucher specimens were deposited at the herbarium of the Shaanxi University of Chinese Medicine (B2019015). The decoction was prepared as previously described by Zhang et al. [18]. The mixture of Lobed Kudzuvine Root 32 g, Baical Skullcap Root 12 g, Golden Thread 12 g and Liquorice Root 8 g was soaked in 512 ml distilled water for 30 min, stewed for 30 min then decoction was collected. Another 512 ml of water was added and stewed for 30 min. Two batches of decoction were mixed and filtered with a 100-mesh sieve. The filtrate was concentrated by a rotary evaporator at 60 °C and then stored at 4 °C before use. The high-performance liquid chromatographic (HPLC) fingerprint analysis of GQD had been done by Zhang et al. and puerarin, berberine, daidzin, liquiritin, coptisine, atrorrhizine, baicalin, palmatine, wogonoside, baicalein, wogonin, ammonium glycyrrhizinate were detected in the extracts [15].

One tablet (0.5 g) of metformin hydrochloride (Freda H20052118) was dissolved in 8 ml of distilled water before use.

Rats of the control group and diabetes group were administered with saline. GQD group was administered with a dose of 4.5 g/kg/d GQD extract which is equal to 15 g/kg/d crude herbs (modified from Zhang et al. [18] and Hang et al. [28]). Metformin group was administered with 300 mg/kg/d [28] metformin solution. Rats in all the groups were intra-gastrically administered with medicine or saline once a day for 6 weeks. The body weight and FBG were measured once a week.

### Sample collection and processing

After 6 weeks of treatment, rats were anesthetized with 2% isoflurane via inhalation (RWD, Shenzhen, China), then blood, liver and pancreas were collected. Rats were euthanized by an overdose of isoflurane (5%). Pancreas and liver tissue were fixed overnight in 4% paraformaldehyde for paraffin sections which were prepared as 3 μm on positively charged glass slides. Part of pancreas tissue was snap frozen in liquid nitrogen and then kept in RNase free tubes for further RNA extraction.

### HE staining

Slides with liver or pancreas paraffin sections were de-paraffinized in xylene and rehydrated through graded ethanol. After being washed with PBS, slides were stained in Harris Hematoxylin solution for 5 min. Followed by 1% acid alcohol differentiation, running tap water rinsing for 15 min, eosin red staining for 10s and distilled water washing. Finally, after dehydration in graded ethanol and xylene for 5 min, slides were mounted and sealed with neutral balsam.

### RNA extraction and qRT-PCR analysis

Total RNA was extracted from pancreas tissue using RNAiso Plus (TaKaRa Biotechnology, Dalian, China) according to the manufacturer’s protocol. Complementary DNA was synthesized from total RNA using Prime Script RT Master Mix (TaKaRa Biotechnology, Dalian, China). Real-time PCR with pancreatic cDNA was performed on a 7500 Real-Time PCR System (Applied Biosystems, CA, USA) using SYBR Green premix EX Taq (TaKaRa Biotechnology, Dalian, China). The results for each specific gene were normalized to the ribosome 18 s RNA gene. The 2^-∆∆Ct^ method [29] was used to calculate the relative fold-changes of each gene. The primers used in this study are shown in Table 1.

**Table 1:** Primer sequences used for qRT-PCR analysis

Gene	Forward primer (5′-3′)	Reverse primer (5′-3′)
18sRNA	GACTCAACACGGGAAACCTCACC	ACCAGACAAATCGCTCCACCAAC
Tp53	GCAGCACAGGAACCTGGAACTG	AGAAGGGACGGAAGATGACAGAGG
Cdk4	GCCTTCCCGTCAGCACAGTTC	GCACAGACATCCATCAGCCGTAC
Smst	AGAACTGCTGTCCGAGCCCAA	AGCTCCAGCCTCATCTCGTC
Irs1	AGCACAAGCCTGTCCTCTCCTAC	AATCTTCGGCAGTTGCGGTATAGC
Gcg	ACCGTTTACATCGTGGCTGGATTG	TCTGGCGTTCTCCTCCGTGTC
Ins1	GGACCCGCAAGTGCCACAAC	TGATCCACAATGCCACGCTTCTG

### Immunohistochemical and immunofluorescent staining

After deparaffinization in xylene and rehydration through graded ethanol, slides were washed with distilled water for 5 min, then incubated with 3% hydrogen peroxide for 20 min to block endogenous peroxidase. Followed by 3 times of 10 min PBS washing, slides were soaked in citrate buffer (pH = 6.0). Antigen retrieval was performed by heating the buffer until boiling and then cooling to 85 °C, this was repeated 3 times. Next, tissues were washed with PBS 3 times, then blocked with 5% BSA for 30 min.

Single labeling was performed by using avidin-biotin complex based DAB staining. Slides with tissue sections were incubated with rabbit anti-insulin polyclonal primary antibody (1:50; BM4310, Boster, Wuhan) or mouse anti-glucagon polyclonal primary antibody (1:200; BM1621, Boster, Wuhan) at 4 °C overnight. Followed by washing, the labeled avidin-biotinylated rabbit IgG kit (SA1022, Boster, Wuhan) or rat IgG kit (SA1021, Boster, Wuhan) were used for amplification of primary antibody binding respectively. Antibody-antigen complexes were visualized via DAB (AR1022, Boster, Wuhan) for 5 min at room temperature. Sections were counterstained with hematoxylin, then dehydrated through graded ethanol and immersed in xylene, finally sealed with neutral balsam.

For double labeling experiments, fluorescent staining was used. The sections were incubated with primary antibodies at 4 °C overnight. Rabbit anti-insulin (1:50; BM4310, Boster, Wuhan) and mouse anti-glucagon (1:200; BM1621, Boster, Wuhan) were used. Sections were then incubated with the secondary antibodies FITC goat anti-rabbit IgG (1:50; BA1105, Boster, Wuhan) and Cy3 goat anti-mouse IgG (1:50; BA1031, Boster, Wuhan) for 1 h in darkness. After rinsing with PBS, the sections were mounted with AR1109 solution (Boster, Wuhan) and sealed. For negative controls, each primary antibody was replaced with normalized serum from the host species before incubation with tissues.

### Glycogen staining

Glycogen was measured using a Glycogen Periodic Acid Schiff (PAS/Hematoxylin) Stain Kit (Solarbio-G1281, Beijing) and the assay was performed according to the manufacturer’s instructions.

### Image acquisition and statistical analysis

HE staining, P.A.S staining and immunohistochemical staining for insulin or glucagon positive cells were imaged by Olympus BX41 microscope. Immunofluorescent staining was scanned by a Zeiss LSM 5 confocal microscope attached to a Zeiss Axiovert 200 M microscope (Carl Zeiss GmbH, Jena, Germany).

The glycogen expression level was evaluated semi-quantitatively by Image J based on the positive P.A.S staining area/islet area. At least 25 randomly selected liver images from 3 to 5 animals of each group were used for P.A.S positive area analysis. Pancreatic immunostaining images were analyzed by Image J software and a total of 17–30 islets from 3 to 5 animals in each group were selected. Cell number was manually counted by Image J multi-point function and positive staining area selection was using the analyze-measure function of Image J, this method was modified from Kilimnik and Marselli [30, 31]. All the images used for analysis were at a magnification of × 400.

Data were reported as the mean ± SEM. The statistical data were processed by GraphPad Prism 8 software. The t-test was used for comparison between the two groups, and the multiple groups variance analysis was used one-way ANOVA test. *P* < 0.05 was considered as significant difference.

### Network pharmacology-based mechanism of GQD and metformin promoted α-cell proliferation

Diabetes and cell proliferation related targets were obtained from OMIM [32] (approved gene was selected) and GeneCards [33] (score > 30) database. Metformin targets were collected from DrugBank [34], Swiss Target Prediction [35] and STITCH [36]. GQD candidate targets were screened from BATMAN [37] (default setting). The diabetes related targets of GQD were screened from TCMSP [38] (the oral bioavailability-OB was set ≥30% and the drug-likeness-DL was set ≥0.18). All the target names were unified in UniProt [39]. Targets protein-protein interaction (PPI) networks were obtained from STRING [40], the plot was constructed using Cytoscape 3.7.2 software. The targets gene function and pathway enrichment analysis was performed by using the Metascape database [41]. In Matascape analysis, Molecular Complex Detection (MCODE) algorithm was applied to identify densely connected network components, pathway and process enrichment analysis has been applied to each MCODE component independently.

## Results

### GQD and metformin increased food intake without affecting the body weight and FBG level of diabetic rats

**Fig. 1:**
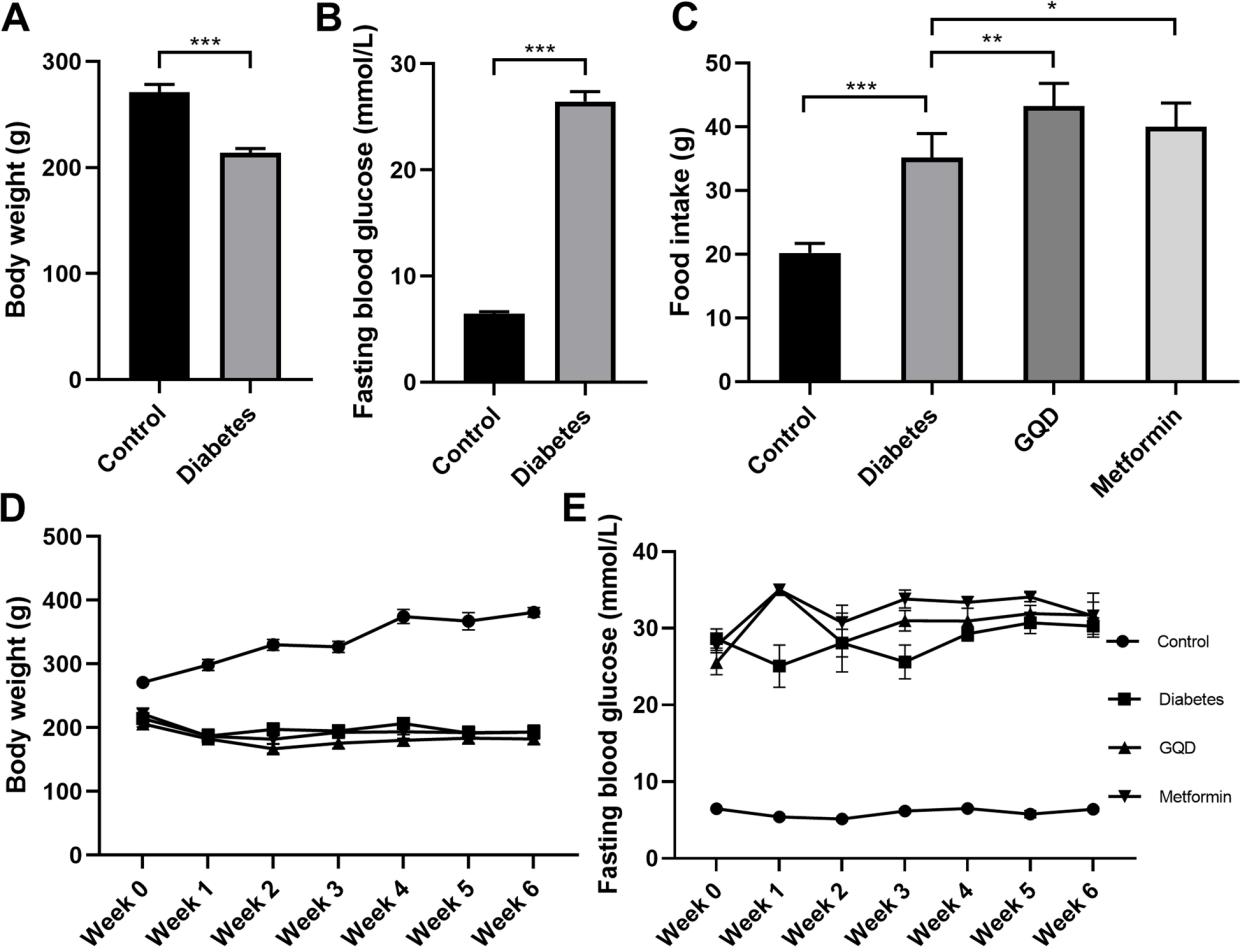
GQD and metformin altered food intake, not FBG of diabetic rats. **A**, STZ injection decreased body weight of the control group; **B**, STZ injection increased FBG of the control group; **C**, Average food intake during medication. Diabetes group had higher food intake than control group (*p <* 0.001) and GQD or metformin increased food intake of diabetic rats (*p <* 0.05); D, Body weight changes during medication; E, FBG changes during medication (* *p <* 0.05, ** *p <* 0.01, *** *p <* 0.001; *n =* 6–24)

After a single dose STZ injection, 80% of rats were successfully induced with diabetes. The body weight of the diabetes group was significantly decreased and the fasting blood glucose (FBG) was significantly higher than the control group (Fig. 1A, Fig. 1B, *p <* 0.001). During the whole treatment, the average food intake per animal in the control group was 20 g/day, while the average food intake in the diabetes group was higher than the control group (*p* < 0.001); the average daily food intake of GQD and metformin group were 43 g/day and 40 g/day, which were significantly increased compared to the diabetes group (Fig. 1C, *p* < 0.05). After 6 weeks of medication, the weight gain was observed in the control group; the body weight of the diabetes group was significantly lower than the control group; the GQD group and metformin group had similar body weight as the diabetes group (Fig. 1D).

During the treatment, the FBG level of the diabetes group was significantly higher than the control group (Fig. 1E, *p* < 0.001). The FBG of the GQD or metformin group was significantly higher than that of the diabetes group in the first week after medication, then remained similar to the diabetes group in the following weeks (Fig. 1E, *p* < 0.05). After 6 weeks of medication, the FBG level of the GQD or metformin group was not significantly different from the diabetes group (Fig. 1E, *p* > 0.05).

### GQD and metformin improved hepatic inflammatory response and decreased liver glycogenesis of STZ induced diabetic rats

**Fig. 2:**
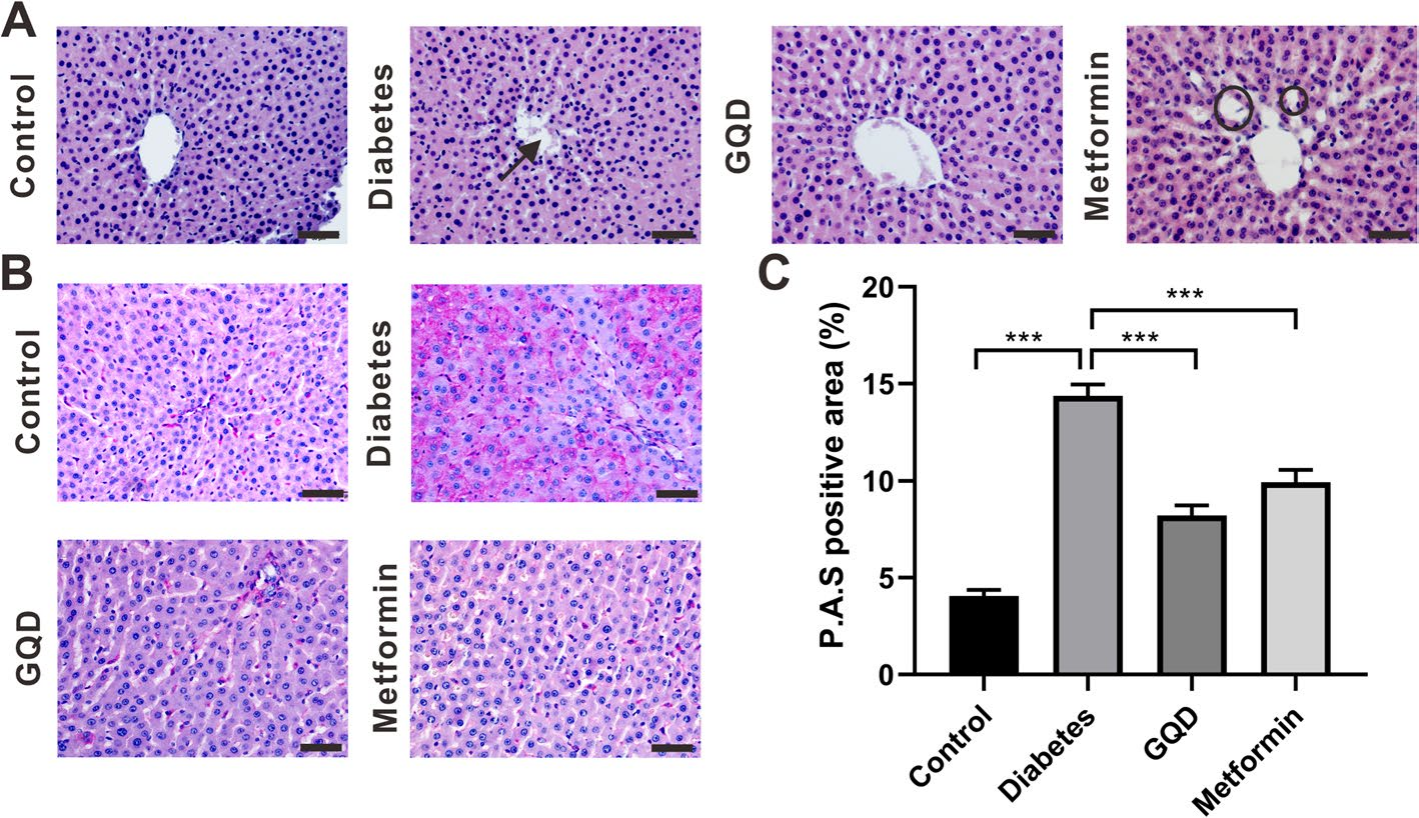
GQD and metformin alleviated hepatic inflammation and decreased glycogenesis of diabetic rats. **A**, HE staining of the liver; **B**, P.A.S staining of the liver; **C**, Percentage of glycogen in the liver. Diabetes significantly increased glycogen level compared to the control (*p <* 0.001), glycogen level in GQD or metformin group was lower than the diabetes group (*p <* 0.001) (*** *p <* 0.001; Scale bar: 50 μm; magnification: 400×; *n =* 25)

The liver is a very important organ to modulate blood glucose level and is the key target of metformin [23]. To detect the liver tissue morphology changes after medication, HE staining was performed. In the control group, the size of hepatocytes was consistent, the hepatic sinusoids were neatly arranged and the hepatic lobule structure was intact (Fig. 2A). In the diabetes group, the central venous endothelium was often found ruptured (Fig. 2A, arrow), along with the hepatocyte death; the hepatic sinusoids structure was disordered in the portal area and inflammatory cell infiltration was also observed. After GQD treatment, the sinusoids were neatly arranged and only a few inflammatory cells in the portal area were observed (Fig. 2A). The central venous structure in the metformin group was intact, inflammatory cells were not observed in the portal area, but some of the hepatic sinusoids were enlarged (Fig. 2A, black circle).

Liver glycogenesis is an important process to regulate FBG level, P.A.S staining was performed to quantify the glycogen level. In the control group, liver glycogen was scattered in the cytoplasm, while diabetic rats had glycogen expressed in the cell plasma and intercellular substance (Fig. 2B). Interestingly, after GQD or metformin treatment, glycogen was randomly distributed and mainly found in the surrounding of the portal triad and portal venule (Fig. 2B). The percentage of the P.A.S positive area was calculated to represent glycogen expression level. The glycogen level of the diabetes group was higher than the control group (*p* < 0.001) and diabetes increased glycogen level was significantly neutralized by GQD or metformin treatment (Fig. 2C, *p* < 0.001), therefore GQD and metformin ameliorate diabetes induced liver glycogenesis.

### GQD and metformin improved islet pathology by increasing the total islet cell number

**Fig. 3:**
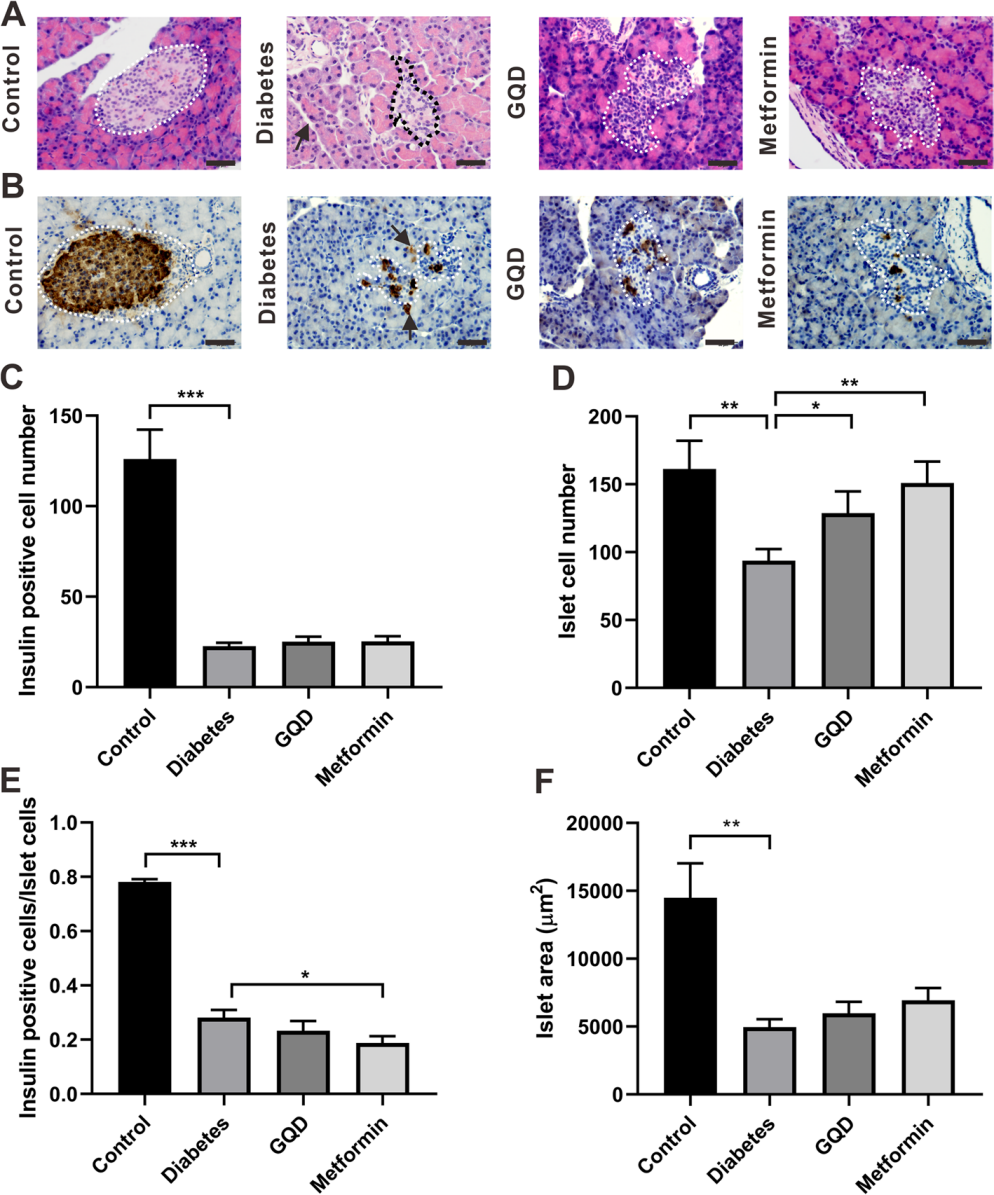
GQD and metformin improved the morphology of diabetic islet by increasing the total islet cell number. **A**, HE staining of the pancreas; **B**, Immunohistochemical staining of insulin in the pancreas; **C**, Insulin positive cell number of islets. Diabetes significantly decreased β-cell number compared to the control (*p <* 0.001); **D**, Total cell number of islets. Diabetes significantly decreased total islet cell number compared to the control (*p <* 0.01) and the total islet cell number in the GQD or metformin group was increased compared to the diabetes group (*p <* 0.05); **E**, Insulin positive cell ratio of islets; **F**, Average islet area of different groups. Diabetes decreased islet area compared to the control group (*p <* 0.01). (* *p <* 0.05, ** *p <* 0.01, *** *p <* 0.001; Scale bar: 50 μm; magnification: 400×; *n =* 17–26)

HE staining was conducted to verify if GQD and metformin can improve STZ induced islet damage. In the control group, pancreatic acinus was tightly arranged with each other and the acinar cells were structurally intact, the islets and acinar cells had recognizable boundaries, the islets were in round or elliptical shape (Fig. 3A, white dotted line) and cells in islet were evenly distributed. In the diabetes group, the space between acinus became wider (Fig. 3A, arrow) and the acinar cells were enlarged, the acinar cells near islets were swollen, the islet shape was irregular and vacuoles were often found in it (Fig. 3A, black dotted line). Compared to the diabetes group, the GQD group had less vacuole in the islet and the boundaries between acinar cells and islets could be observed (Fig. 3A). Compared to the diabetes group, the metformin group had a larger islet area and stable islet morphology (Fig. 3A, white dotted line), the acinar cells around the islets were neatly arranged.

To investigate whether metformin or GQD treatment changed the islet β-cell mass, pancreatic tissues were immuno-histochemically stained with insulin. In the control group, islets were in round or elliptical shape and the insulin positive cells were dominantly expressed in the center of the islet (Fig. 3B, dotted line). In the diabetes group, islets were in irregular shape and insulin positive cells were dispersedly expressed in the islet (Fig. 3B, dotted line), some cells outside of the islet were also positively stained with insulin (Fig. 3B, black arrow). After GQD or metformin treatment, although the insulin positive cells were still scattered in the islet, the shape of the islet was enlarged compared to the diabetes group, the cells in the islet were tightly packed (Fig. 3B, dotted line).

In the diabetes group, there was an 80% decrease in the insulin positive cell number compared to the control group (*P <* 0.001), neither GQD nor metformin treatment increased insulin cell expression compared to the diabetes group (Fig. 3C). STZ reduced the total islet cell number of the control rats (*P <* 0.01), GQD treatment increased the total islet cell number of the diabetic rats (Fig. 3D, *P <* 0.05). Compared to the diabetes group, metformin treatment significantly increased total islet cell number (Fig. 3D, *p <* 0.01), which lead to a significant decrease in β-cell to total islet cell ratio (Fig. 3E, *P <* 0.05). The islet area in the diabetes group shrank to a smaller size compared to the control group (Fig. 3F, *P <* 0.01). GQD or metformin treatment slightly increased the islet area compared to the diabetes group, but the difference was not statistically significant (Fig. 3F, *P >* 0.05). Taken together, both metformin and GQD treatment improved the islet morphology of STZ induced diabetes by increasing the total cell number.

### GQD and metformin increased the islet α-cell number of STZ induced diabetic rats

**Fig. 4:**
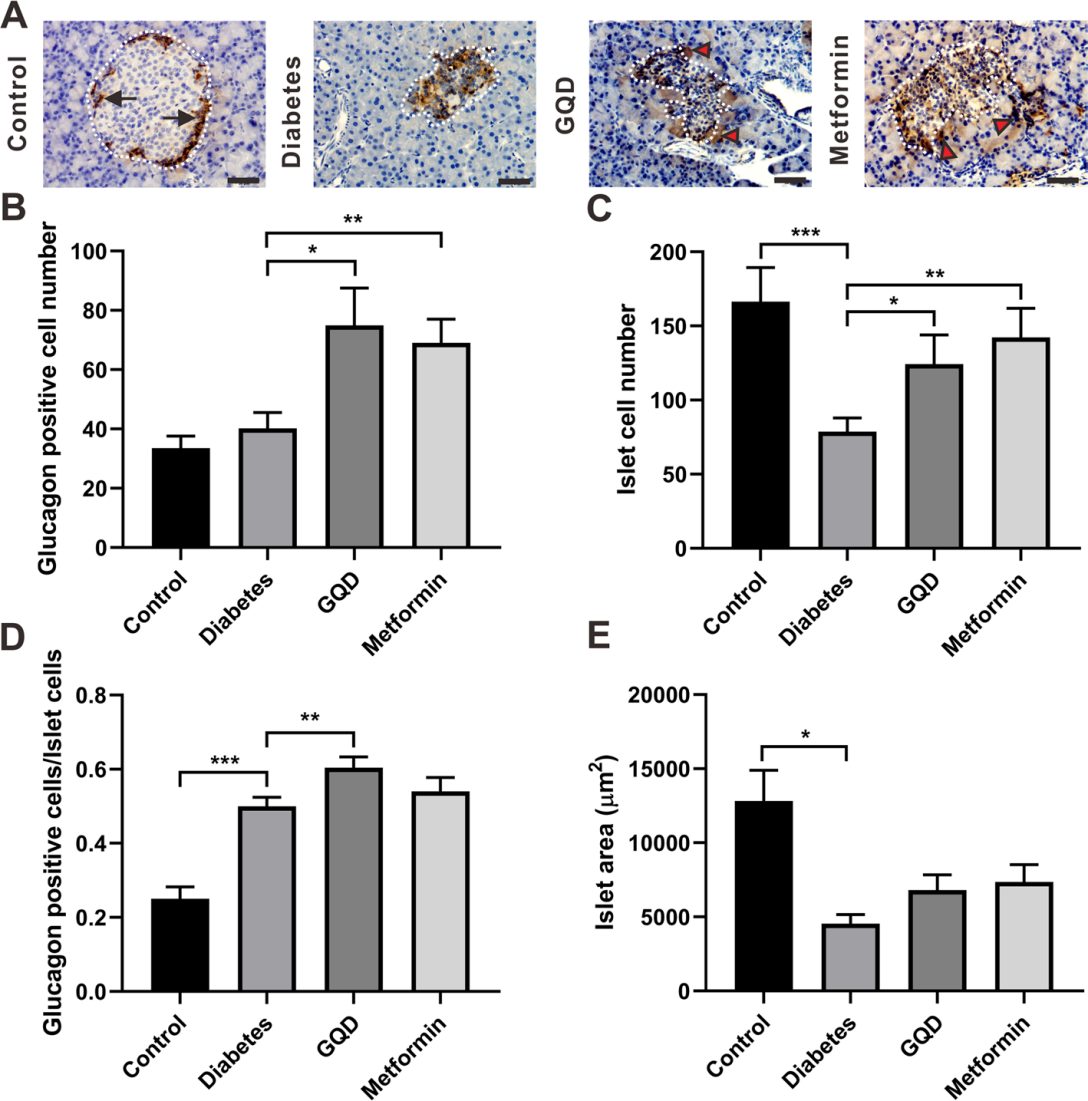
GQD and metformin significantly increased glucagon positive cell number of STZ induced diabetic rats. **A**, Immunohistochemical staining of glucagon in the pancreas; **B**, Glucagon positive cell number of islets. The glucagon cell number of the GQD or metformin group was increased compared to the diabetes group (*p <* 0.05); **C**, Total cell number of islets. The diabetes group had a lower total islet cell number compared to the control (*p <* 0.001) and GQD or metformin treatment increased the total islet cell number of diabetic rats (*p <* 0.05); **D**, Glucagon positive cell ratio of islets. The glucagon cell ratio was higher in the diabetes group than in the control group (*p <* 0.001), GQD treatment increased the total glucagon cell ratio of diabetic rats; **E**, Average islet area of different groups. Diabetes decreased islet area compared to the control group (*p <* 0.05). (* *p <* 0.05, ** *p <* 0.01, *** *p <* 0.001; Scale bar: 50 μm; magnification: 400×; *n =* 22–30)

To detect whether GQD and metformin increased total islet cell number by promoting α-cell proliferation, pancreatic tissue was stained for glucagon. In the control group, the glucagon positive cells were localized at the rim of the islets, which was as expected in healthy animals (Fig. 4A, arrow). In the diabetes group, the islets were in irregular shape and the glucagon positive cells were randomly distributed in the islet residue (Fig. 4A, dotted line). In GQD and metformin groups, the glucagon positive cells were randomly localized in the islet and some glucagon positive signals were also found in acinar cells that surrounded the islet (Fig. 4A, red arrow head).

The glucagon positive cell numbers of the control group and diabetes group were not significantly different (*P >* 0.05); the glucagon positive cell number of the GQD or metformin group increased by more than 70% compared to the diabetes group (Fig. 4B, *p <* 0.05). The total islet cell number of the diabetes group was lower than the control group (*P <* 0.01) and GQD or metformin treatment increased the total islet cell number of diabetic rats (Fig. 4C, *P <* 0.05). The glucagon cell ratio of the diabetes group was higher than the control group (*p <* 0.001) and the glucagon cell ratio of the GQD group was significantly increased compared to the diabetes group (Fig. 4D, *P <* 0.01). The islet area was decreased in the diabetes group when compared to the control (Fig. 4E, *p <* 0.05). These results indicate that both GQD and metformin provided partial recovery of diabetes induced islet damage by increasing islet α-cell number.

### GQD and metformin increased islet cell mass of STZ induced diabetes rats by boosting islet α-cell, not β-cell proliferation

**Fig. 5:**
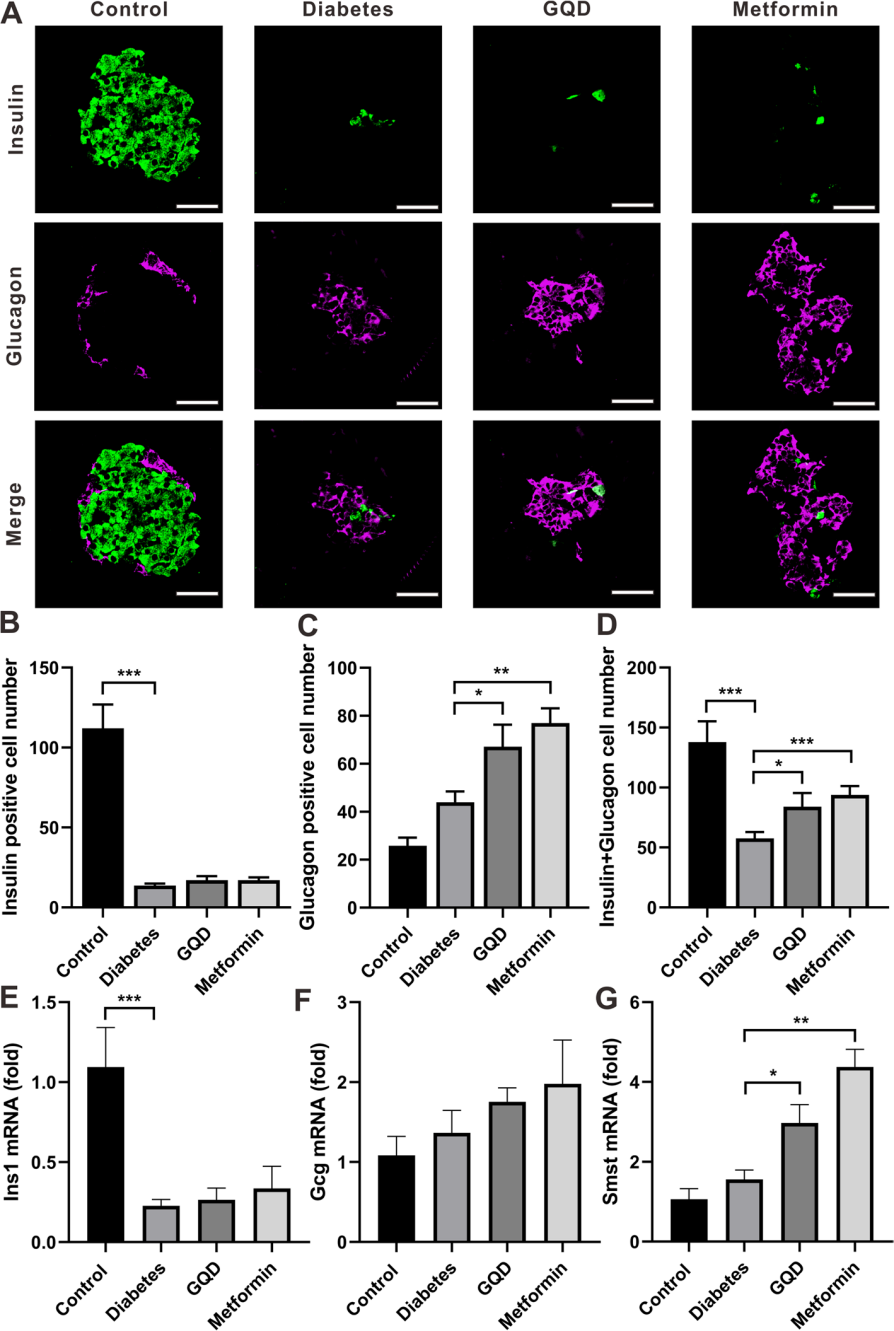
GQD and Metformin significantly increase islet α-cell mass and Smst mRNA expression of diabetic rats. **A**, Immunofluorescent double labeling of insulin and glucagon in the pancreas; **B**, Insulin positive cell number of islets. Diabetes significantly decreased insulin cell number compared to the control (*p <* 0.001); **C**, Glucagon positive cell number of islets. GQD or metformin increased the glucagon cell number of diabetic rats (*p <* 0.05); **D**, Insulin plus glucagon positive cell number. The diabetes group had a lower cell number compared to the control (*p <* 0.001) and the GQD or metformin group had a higher cell number than the diabetes group (*p <* 0.05); **E**, Ins1 mRNA expression. Diabetes suppressed the Ins1 expression of the control; **F**, Gcg mRNA expression; G, Smst mRNA expression. The Smst expression level in GQD or metformin group was higher than the diabetes group (*p <* 0.05). (* *p <* 0.05, ** *p <* 0.01, *** *p <* 0.001; Scale bar = 50 μm; magnification: 400×; B *n =* 22–24; E, *n =* 4)

To verify whether GQD and metformin increased the islet cell mass of diabetic rats via promoting the proliferation of islet α-cell, both insulin and glucagon were immuno-fluorescently stained in pancreatic tissue. In the islet of the control group, both insulin and glucagon positive cells were detected, insulin positive cells were dominantly expressed in the islet center which was surrounded by some glucagon positive cells (Fig. 5A). In the islet of the diabetes group, there were a few insulin positive cells randomly distributed, with some glucagon positive cells aggregated in the center (Fig. 5A). The GQD and metformin groups represented similar insulin and glucagon co-expression pattern as the diabetes group (Fig. 5A).

The average insulin positive cell number in the diabetes group was much lower than the control group (*p <* 0.001) and the GQD or metformin treatment did not cause a significant difference in insulin cell number compared to the diabetes group (Fig. 5B, *P >* 0.05). Nevertheless, the glucagon positive cell number in the diabetes group was similar to the control group (*P >* 0.05), both GQD and metformin significantly increased the glucagon cell number of the diabetic rats (Fig. 5C, *p <* 0.05). When insulin and glucagon positive cell numbers were added together, the cell number in the diabetes group was dramatically decreased compared to the control group (*p <* 0.001), both GQD and metformin groups had higher cell numbers than the diabetes group (Fig. 5D, *P <* 0.05). In summary, GQD or metformin treatment increased the islet cell number of STZ induced diabetic rats by boosting islet α-cell proliferation.

To investigate the gene expression pattern of pancreatic hormones (insulin, glucagon and somatostatin) that secrete by three major islet cells (α-cell, β-cell and δ-cell), insulin 1 (Ins1), glucagon (Gcg) and somatostatin (Smst) gene expression were tested. Diabetes caused the decrease in normal Ins1 expression (*P <* 0.001), GQD and metformin did not change the Ins1 expression pattern of diabetic rats (Fig. 5E, *P >* 0.05). GQD or metformin increased the Gcg mRNA expression of diabetic rats but the difference was not statistically significant (Fig. 5F, P > 0.05). Diabetes did not change Smst RNA level compared to the control (P > 0.05), but GQD or metformin significantly increase Smst mRNA level as compared to the diabetes group (Fig. 5G, *P* < 0.05).

### Network pharmacology-based mechanism of GQD and metformin boost islet α-cell proliferation

#### GQD and metformin targets prediction

To investigate the potential targets of GQD and metformin that exert cell proliferation role in STZ induced diabetic rats, 305 diabetes targets and 763 cell proliferation targets were screened from GerneCards and OMIM. After comparing the diabetes related targets of GQD with cell proliferation targets, 4 potential targets (PPARG, TNF, INSR, CDK4) were selected for GQD (Table 2). After comparing the diabetes related targets of metformin with the targets of diabetes and cell proliferation, 4 targets (TP53, IGF1R, IRS1, LEP) were selected for metformin (Table 2).

**Table 2:** Targets of GQD and metformin

GQD targets for diabetes	GQD targets for diabetes and cell proliferation	Metformin targets for diabetes	Metformin targets for diabetes and cell proliferation
PPARG	PPARG	IGF1R	TP53
Ptpn1	TNF	IRS1	IGF1R
TNF	INSR	LEP	IRS1
KCNJ11	CDK4	SLC2A4	LEP
AKR1B1		TP53	
DPP4			
GSK3B			
INSR			
GAA			
PYGM			
PPARD			
CDK4			
MGAM			

#### Metformin and GQD targets PPI interaction

**Fig. 6:**
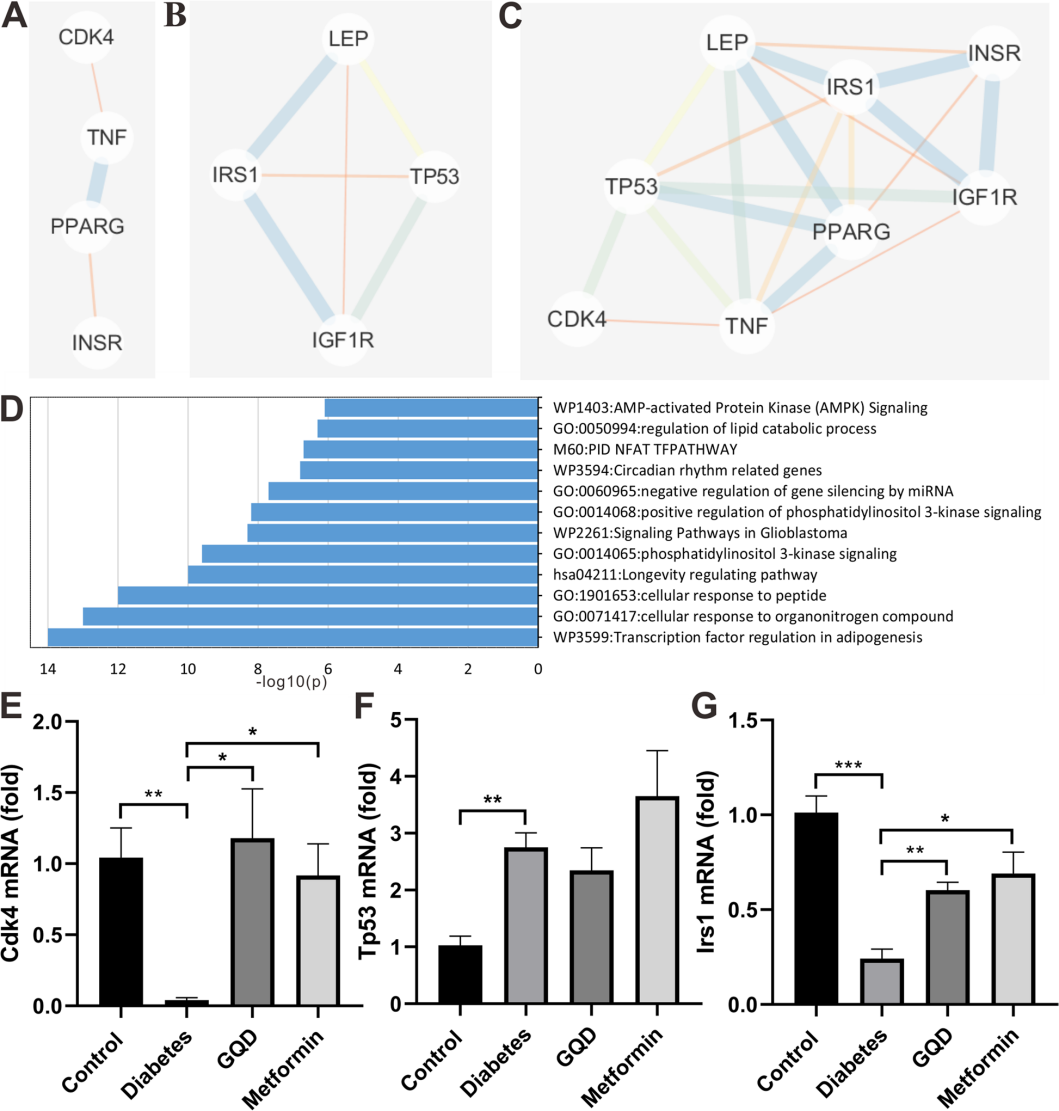
Network based mechanism of GQD and metformin boost islet α-cell proliferation. **A**, PPI network of GQD targets on diabetes and cell proliferation; **B**, PPI network of metformin targets on diabetes and cell proliferation targets; **C**, PPI network of GQD and metformin targets on diabetes and cell proliferation; **D**, Metformin and GQD cell proliferation targets gene pathway and process enrichment analysis; **E**, RNA expression of Cdk4. Diabetes suppressed the Cdk4 expression compared to the control group (*p <* 0.01). The Cdk4 expression was increased in GQD or metformin group compared to the diabetes group (*p <* 0.05); **F**, RNA expression of Tp53. Diabetes increased the Tp53 expression of the control rats; **G**, RNA expression of Irs1. Diabetes suppressed the Irs1 expression compared to the control group (*p <* 0.001). The Irs1 expression was increased in GQD or metformin group compare to the diabetes group (*p <* 0.05) (* *p <* 0.05, ** *p <* 0.01, *n =* 4)

To detect the protein-protein interaction (PPI) of screened targets for diabetes and cell proliferation, GQD (PPARG, TNF, INSR, CDK4) or metformin (TP53, IGF1R, IRS1, LEP) targets were analyzed through STRING. CytoScape 3.7.2 was used for the analysis and construction of the targets PPI network of GQD and metformin. The GQD targets showed a liner PPI network (Fig. 6A) and metformin targets showed a multilateral interaction (Fig. 6B). After pooling the screened targets of diabetes and cell proliferation from GQD and metformin, the PPI network showed a linear interaction from CDK4 to IRS1 (Fig. 6C).

### GQD and Metformin targets gene function and pathway enrichment analysis

To understand the targets gene function and metabolic pathway enrichment, TP53, IGF1R, IRS1, LEP, PPARG, TNF, INSR, CDK4 were analyzed by Metascape database [41]. The result showed that TP53, IRS1, TNF and IGF1R were the best-scoring components. The -log10 value (*P*-value) provided an enrichment score that indicated the significance of the gene pathway and process enrichment. The targets are mainly involved in transcription factor regulation in adipogenesis, cellular response to organonitrogen compound, cellular response to peptide, longevity regulation pathway (Fig. 6 D).

### The mRNA expression level of the predicted targets

To test the expression pattern of prominent proliferation targets gene, quantitative real-time PCR analysis for cyclin-dependent kinase 4 (Cdk4), tumor protein p53 (Tp53) and insulin receptor substrate (Irs1) were performed. Compared to the control group, diabetes induced a dramatic decrease in Cdk4 expression (*P* < 0.01), GQD and metformin significantly increased the Cdk4 level of diabetic rats (Fig. 6 E, *P* < 0.05). Compared to the control group, the Tp53 mRNA level was elevated in the diabetes group (*P <* 0.01), GQD and metformin group had similar Tp53 expression as the diabetes group (Fig. 6 F, *P >* 0.05). The Irs1 gene expression was decreased in the diabetes group when compared to the control (*P <* 0.001), both GQD and metformin can increase the Irs1 expression level which was suppressed by diabetes (Fig. 6 G, *P <* 0.05). Diabetes suppressed Cdk4 and Irs1 gene expression that was elevated by GQD and metformin indicated these two genes might be the key targets for α-cell proliferation.

## Discussion

**Fig. 7:**
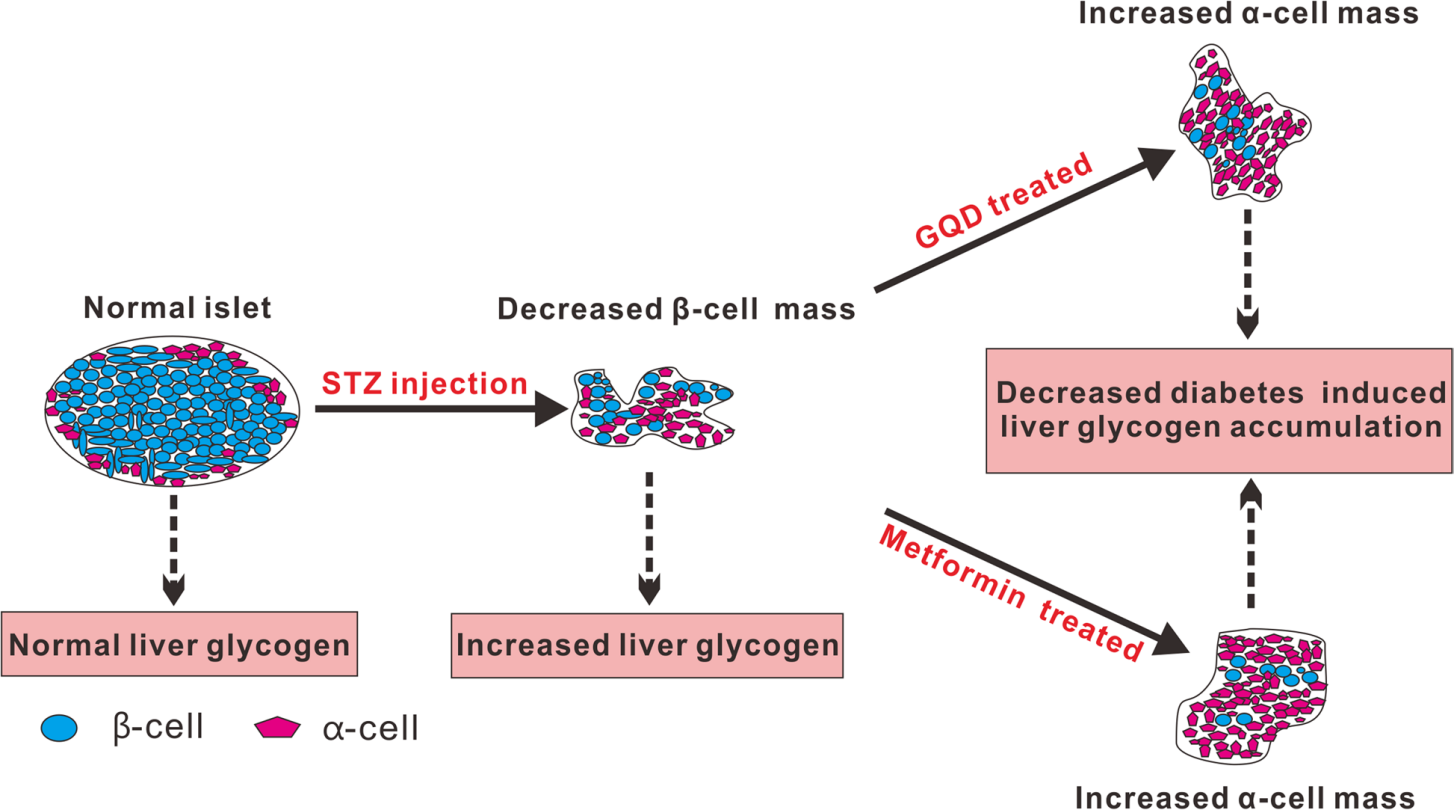
Hypothesis of the relationship of liver glycogen and islet α-cell number

Reductions in β-cell mass and abnormalities of β-cell mass are necessary for the development of hyperglycemia [9]. Deficit of β-cell mass is not only a hallmark of T1D [25] but also found in up to 63% of long standing T2D patients [42]. Metformin and traditional Chinese medicine GQD were commonly used to treat newly diagnosed T2D patients in China and some Asian countries. With the progress of T2D, patients will gradually develop β-cell deficiency or even apoptosis [7, 8], after islet β-cell reduction whether these two medicines can alleviate diabetes or not is still elusive. This research used STZ to induce β-cell deficiency caused diabetes then treated the diabetic rats with GQD or metformin. The results from this research demonstrated that GQD and metformin stimulated islet α-cell proliferation and glycogenolysis of the β-cell deficiency caused diabetes (Fig. 7).

Since STZ specifically destroys the islet β-cells without affecting the morphology of other cells in the pancreas, rats were injected with a single dose of STZ to induce islet β-cell deficiency caused diabetes [12]. GQD dose that equals 4.95, 11.55 and 18.15 g/kg of crude herbs significantly decreased FBG of diabetic rats and GQD at 18.15 g/kg had the potential to regulate insulin secretion [18]. According to the GQD raw materials used in the clinic, T2D rats were administered with high, middle or low doses of GQD and the results indicated that GQD at 13.365 g/kg displayed the greatest efficacy [28]. GQD extraction equal to 13.36 g/kg of crude herbs has also been reported to protect pancreatic β-cells [43]. Therefore, higher dose GQD has better effects on islet cells. This work focuses on the effects of GQD on β-cell deficiency induced diabetes, the GQD dose equal to 15 g/kg of crude herbs was chosen as modified the formula from Hang et al. [28]. In previous reports, metformin doses used for diabetes treatment ranges from 200 mg/kg/d [18], 300 mg/kg/d [28], 420 mg/kg/d [44] to 500 mg/kg/d [45]. For low dose STZ induced diabetic rats, 200 mg/kg/d metformin was sufficient to reduce FBG [46]. In this research due to the massive loss of β-cells, 300 mg/kg/d metformin was chosen as it was reported to improve the islet morphology of the STZ induced diabetic rat [28, 43].

Even though GQD and metformin have been reported to decrease FBG level in diabetic rats. In this research, GQD and metformin administration did not significantly change the FBG level of β-cell reduction induced diabetic rats (Fig. 1E). That might be due to the massive β-cell loss caused by STZ injection. The immunohistochemistry results demonstrate that when islet β-cells were dramatically destroyed, continued administration of GQD or metformin promotes the total islet cells growth without affecting the β-cell number in the islets (Fig. 3). Further glucagon immunochemistry staining indicated that both α-cells and total islet cell numbers were increased in GQD and metformin groups (Fig. 4 A-Fig. 4 C). Double staining results proved that the increase in pancreatic islet cell number was due to the proliferation of islet α-cells since when α-cells were counted alone or summed up with β-cells the cell number in GQD and metformin groups were significantly higher than in the diabetes group (Fig. 5 A-Fig. 5 D).

Islet β-cells secreted insulin and α-cells secreted glucagon working together to balance the blood glucose level [3]. It has been proved that α-cell signals stimulate insulin secretion from the neighboring β-cell [47]. A recent study showed that glucagon increases insulin levels by stimulating insulin secretion [48], indicating that the α-cell proliferation promoted by GQD and metformin may modulate insulin secretion by increasing glucagon level in the islet. Moreover, the presence of α-cells has a positive effect on glucose-stimulated insulin secretion (GSIS) from pancreatic β-cell [49]. In islet, endogenous glucagon potentiates insulin secretion during hyper-glycemia [50].

In the islet, the insulin producing β-cells crosstalk with the glucagon producing α-cells and somatostatin producing δ-cell to maintain the stable blood glucose level [50]. Pancreatic δ-cell derived somatostatin serves a largely paracrine role within the islet as a local regulator of insulin and glucagon release. As an important regulator of glucose homeostasis, somatostatin plays a role of an inhibitor for glucagon release [51]. When islet α-cell mass was promoted by GQD and metformin administration, the mRNA expression of Smst was also increased (Fig. 5 G). The overexpression of Smst could produce more somatostatin that counteracts with the α-cells to suppress too much glucagon secretion. This result is similar to the finding that β-cell ablation was associated with a significant increase in both glucagon and somatostatin expression in zebrafish [52].

Network based pharmacology is efficient in assessing the mechanism and action of TCM herbs or drugs. To understand the mechanism of GQD and metformin induced α-cell proliferation in diabetic rats, Metascape database was chosen for screening the cell proliferation related targets, 4 potential targets (TP53, IRS1, TNF and IGF1R) were the best-scoring components. Cyclin-dependent kinase 4 (CDK4) and insulin receptor (INSR) were indirectly connected at two ends of the GQD PPI network (Fig. 6A). CDK4 plays a key role in mammalian cell proliferation, so it might be the key target for α-cell proliferation. Insulin receptor substance 1 (IRS1) is a downstream protein of insulin receptor (INSR) that acts as a cellular adaptor molecule to mediate metabolic actions after INSR activation, such as glucose uptake, lipid metabolism and cell proliferation [53]. PPI network of GQD and metformin indicates a close connection of CDK4 to TP53 (Fig. 6C), so Tp53, Irs1 and Cdk4 were selected for qRT-PCR analysis. The gene expression study showed that both GQD and metformin can increase Cdk4 and Irs1 gene expression in the diabetic rats, but did not change the Tp53 expression that was up-regulated by β-cell deletion (Fig. 6 E-Fig. 6 G). These results from targets analysis provide a source to further study the mechanism of GQD and metformin induced α-cell proliferation.

STZ injection significantly increased FBG due to the damage of islet β-cells, which in turn affects the metabolism of glucose in the liver, causing pathological damage to liver tissue. Glycogen storage is important to maintain FBG level, counter-regulation of glucagon is essential for diabetic patients who have limited residual functional β-cell mass. Glucagon can increase blood glucose level by stimulating glycogenolysis in the liver [54]. Therefore, GQD and metformin stimulated α-cell proliferation may increase glucagon secretion, leading to decreased liver glycogen storage in diabetic rats (Fig. 2 B, Fig. 2 C), at the same time improving the liver pathological state (Fig. 2A).

Although GQD and metformin did not suppress the FBG level after islet β-cells were extremely decreased, they are still promising medicines in treating diabetes. Firstly, GQD and metformin could be used as effective medications to treat patients in the early stage of T2D or pre-diabetes [55, 56]. Furthermore, the proliferation of α-cells stimulated by GQD and metformin provided an important source for α-cell to β-cell reprogramming. Recently, α-cells were proposed as a good source for a new β-cells generation that can replace the lost or dysfunctional β-cells in T1D and T2D [57]. Islet α-cells and β-cells have the same developmental origin and also express similar genes [58]. Reprogramming strategies for the generation of insulin producing β-cells from α-cells have been investigated [59]. When using GQD for diabetes, due to its limited oral bioavailability as a plant-based drug [60], a better drug delivery system such as microparticulate [61] and nanoparticles [62] might be needed in the future research.

## Conclusions

Massive islet β-cell reduction induced hyperglycemia and glycogenesis. GQD and metformin stimulated islet α-cell increase and glycogenolysis of β-cell deficiency caused diabetes. GQD and metformin promote α-cell proliferation by restoring Cdk4 and Irs1 genes expression. Our results provide a resource for diabetes gene therapy when using reprogramming strategies to generate β-cells from α-cells.

## Data Availability

All data generated or analyzed during this study are included in this article.
